# VIS-NIR, SWIR and LWIR Imagery for Estimation of Ground Bearing Capacity

**DOI:** 10.3390/s150613994

**Published:** 2015-06-15

**Authors:** Roemi Fernández, Héctor Montes, Carlota Salinas

**Affiliations:** 1 Centre for Automation and Robotics (CAR) CSIC-UPM, Ctra. Campo Real, Km. 0.2, La Poveda, Arganda del Rey, Madrid 28500, Spain; E-Mails: hector.montes@car.upm-csic.es (H.M.); carlota.salinas@car.upm-csic.es (C.S.); 2 Faculty of Electrical Engineering, Technological University of Panama, Panama City 0819, Panama

**Keywords:** ground bearing capacity, VIS-NIR, LWIR, SWIR, multispectral, soil moisture, optical filters, penetrometer, soil compaction

## Abstract

Ground bearing capacity has become a relevant concept for site-specific management that aims to protect soil from the compaction and the rutting produced by the indiscriminate use of agricultural and forestry machines. Nevertheless, commonly known techniques for its estimation are cumbersome and time-consuming. In order to alleviate these difficulties, this paper introduces an innovative sensory system based on Visible-Near InfraRed (VIS-NIR), Short-Wave InfraRed (SWIR) and Long-Wave InfraRed (LWIR) imagery and a sequential algorithm that combines a registration procedure, a multi-class SVM classifier, a K-means clustering and a linear regression for estimating the ground bearing capacity. To evaluate the feasibility and capabilities of the presented approach, several experimental tests were carried out in a sandy-loam terrain. The proposed solution offers notable benefits such as its non-invasiveness to the soil, its spatial coverage without the need for exhaustive manual measurements and its real time operation. Therefore, it can be very useful in decision making processes that tend to reduce ground damage during agricultural and forestry operations.

## 1. Introduction

The utilisation of wheeled and tracked vehicles is a widespread practice in agriculture and forestry applications [1]. However, operations with this kind of heavy machines can cause compaction and rutting, producing severe soil damage [2,3,4]. Soil is one of the most fundamental components for supporting life on Earth [5]. When soil is compacted and/or rutted, its porosity decreases, and consequently, the amount of oxygen that is required for a healthy function of plant roots [6]. In addition, if pore diameters become smaller than root tips, roots will have to become thicker to exert more force to penetrate soil, what can delay their growth or even make growth cease [7]. Soil thermal regime, water-air relationship, as well as the microbiological activity may also be drastically disturbed by compaction or rutting, significantly reducing the potential for growth and survival of plants [8,9]. Estimation of ground bearing capacity becomes then an important factor to be considered for achieving efficient and environmental friendly propulsion of both manned and autonomous agricultural and forest vehicles.

Ground bearing capacity can be defined as the ability of the soil to carry a certain weight without being damaged [10]. In forestry, ground bearing capacity is usually considered as the maximal allowable wheel contact pressure [11]. Nevertheless, the actual wheel contact pressure is difficult to calculate because the true contact area depends on tyre and soil properties [12]. For that reason, ground bearing capacity is considered, in most situations, as a guideline parameter only, and so far there is no standard procedure for its estimation [13]. Penetrometers are the only available instruments for obtaining an empirical measure of soil strength that allows comparisons between different soils [14]. The simplest penetrometer is a hand-held device that is pushed downward at a constant rate through a specified increment of soil depth. The American Association of Agricultural Engineers specified a standard penetrometer design that provides a measurement called the cone index, which is calculated by dividing the insertion force by the area of the circular cone base [15]. Although standardised, cone penetrometers exhibit several drawbacks. A cone penetrometer should be inserted in the terrain at a constant speed, which can be difficult to achieve manually. In addition, the measurement is made at a single discrete point, being necessary to carry out numerous measurements for mapping an entire field [16]. These facts make that the sampling of a terrain with a cone penetrometer turns into a cumbersome and time-consuming technique.

On the other hand, the capability of the soil to bear an specific weight without suffering from compaction and/or rutting is predominantly determined by the soil type and the water content [17,18]. Since soil water is the only parameter varying over the time, its estimation could contribute significantly to make strategic decisions for ground protection during mechanical operations.

Several techniques have been proposed for measuring the soil water content, including direct, indirect and remote sensing methods [19,20,21]. Direct methods may be regarded as those methods wherein water is removed from a soil sample by evaporation, leaching or chemical reaction. The soil water content is then determined considering the mass of water removed and the mass of the dry soil [19]. Indirect methods comprise measurement of some property of the soil that is modified by soil water content [20]. Remote methods rely on the measurement of electromagnetic energy that has either been reflected or emitted from the soil surface [21].

The thermogravimetric technique is the oldest established and the only truly direct method for determining the total moisture content of soils. The method implies to oven-dry a soil sample of known weight at a constant temperature of 105 °C. After that, the sample is cooled and reweighted. The water content of the sample is then given by the mass of water per unit mass of dry soil [14,19,21].

The most common indirect methods for estimating the soil water content are the neutron scattering, the use of porous blocks, the dielectric measurements by means of capacity sensors and Time-Domain Reflectometry (TDR) [22,23]. The neutron scattering technique estimates the amount of water in a volume of soil by measuring the amount of hydrogen present. A radioactive source (^241^Am) is inserted into the soil, from which fast neutrons are emitted. When these particles collide with the hydrogen atoms present in the water molecules, they lose energy and slow down. Therefore, the total amount of water in the soil is measured taken into account the rate of neutron slowdown [14,21,24]. Porous blocks are made of materials such as gypsum, ceramic, nylon and fiberglass. When these blocks are buried in soil, their water content comes to potential equilibrium with that of the soil. Then, different properties of the block which are affected by its water tension may be measured. Some of the more common types of porous block are the electrical resistance blocks and the thermal dissipation blocks. On the former type, the resistance is affected by the water content of the block, which is a function of the soil water tension. On the latter type, the heat dissipation measured in the soil is correlated to the soil moisture content [24,25]. Based on the soil dielectric constant, the soil moisture content may also be determined by measuring the capacitance between two electrodes implanted in the soil [26,27,28]. The TDR determines the dielectric constant of the soil by measuring the propagation of an electromagnetic pulse, which is launched along a waveguide inserted in the soil. The dielectric constant measured by the TDR constitutes a good approximation of the soil water content [29,30].

Although non-invasive techniques have attracted a lot of attention in the last decade, they are not well established yet. Among these techniques the NIR spectroscopy, the use of Ground Penetrating Radars (GPRs) and remote sensing are noteworthy. In [31] a fiber-type visible and NIR spectrophotometer is proposed for the on-line measurement of soil water content, taking into consideration the fundamental property of water to absorb light energy strongly at NIR bands. The sensor is attached to a subsoiler installed in a tractor, and its performance is improved if soil-to-sensor distance variation is minimised. In [32] NIR diffuse reflectance spectroscopy is utilised for real-time measurement of the soil water content. The device uses a tungsten halogen bulb to illuminate the soil and an optic to direct reflected light into a fiber optic for transmission to the spectrometer. Measurements are carried out through a sapphire window durable enough to withstand continuous contact with the soil. On the other hand, two different approaches are considered for determining the soil surface water content with GPRs. The first one derives the soil surface water content from the ground-wave propagation velocity [33,34], while the second uses the surface reflection coefficient method [35,36]. Airbone and spaceborne remote-sensing based on either passive microwave radiometry or active radar instruments are very promising for estimating soil surface water content over large areas, but their major limitations are the unknown within-pixel heterogeneity and the usually resulting poor agreement with calibrating and gravimetric sampling [37,38]. It is also worthwhile to mention that in [39], visible, multispectral, SWIR, MidWave InfraRed (MWIR), LWIR, polarisation and stereo sensors are experimentally tested and compared for mud detection. Authors conclude that none of these passive sensors alone can detect mud in a robust manner and recommend the combination of colour, stereo and polarization for achieving the stated purpose. Table 1 summarises the main advantages and drawbacks of the techniques mentioned above for measuring the soil water content.

**Table 1:** Main advantages and disadvantages of methods for estimating soil water content.

Techniques		Advantages	Disadvantages
**Direct method**	Thermogravimetric technique	▪ Inexpensive ▪ Simple ▪ Highly accurate ▪ Not dependent on the soil type	▪ Time consuming ▪ Destructive to the soil ▪ Labour-intensive ▪ Difficult in rocky soil ▪ Without automation possibilities
**Indirect methods**	Neutron scattering	▪ Rapid (response time: 1 or 2 min) ▪ Accurate ▪ Repeatable measurement of soil	▪ The use of radioactive material requiring a licensed and extensively trained operator ▪ The high equipment cost ▪ Extensive calibration
	Porous blocks	▪ Quick ▪ Repeatable ▪ Relatively inexpensive	▪ The blocks do not work well in coarse-textured or saline soils. ▪ They are destructive to the soil. ▪ Accuracy is poor. ▪ The blocks need to be soaked in water for several hours before installing them in the field. ▪ Response time: 2 or 3 h
	Capacity sensors	▪ Instantaneous	▪ Intense calibration is required.
	TDR	▪ Independent of soil texture, temperature, and salt content ▪ Possible to perform long-term *in situ* measurements ▪ No calibration is needed ▪ Can be automated	▪ Costly ▪ Destructive to the soil.
**Remote methods**	NIR spectroscopy	▪ Real time ▪ Non-invasive	▪ Dependence on surface roughness
	GPRs	▪ Real time ▪ Non-invasive ▪ High resolution ▪ High speed data acquisition ▪ No limitations because of ambient conditions ▪ Non-destructive method	▪ The most significant performance limitation of GPR is in high-conductivity materials such as clay soils and soils that are salt contaminated. ▪ Interpretation of radargrams is generally non-intuitive. ▪ Considerable expertise is necessary to effectively design, conduct, and interpret GPR surveys. ▪ Relatively high energy consumption can be problematic for extensive field surveys.
	Microwave	▪ Real time ▪ No disturbance of site ▪ No calibrations are required	▪ Not as accurate as direct methods ▪ Susceptible to surface roughness and vegetation ▪ Complex equipment ▪ Costly

This paper proposes an arrangement composed of a SWIR camera, a LWIR camera and a VIS-NIR system that allows interchanging two band-pass optical filters with centre wavelengths of 624 nm and 950 nm and an associated algorithm for the estimation of the ground bearing capacity. The presented approach represents a non-contact method that enables to acquire measurements before traversing the field. In this way, no disturbance of the site will be produced and ground damages can be significantly reduced. The rest of the paper is organised as follows: Section 2 presents the sensory system that has been designed and tested, as well as the algorithm implemented for the estimation of the ground bearing capacity. Section 3 describes the results obtained from the different experimental tests that have been carried out. Section 4 discusses the main results, while Section 5 summarises major conclusions and future research directions.

## 2. Materials and Methods

**Figure 1:**
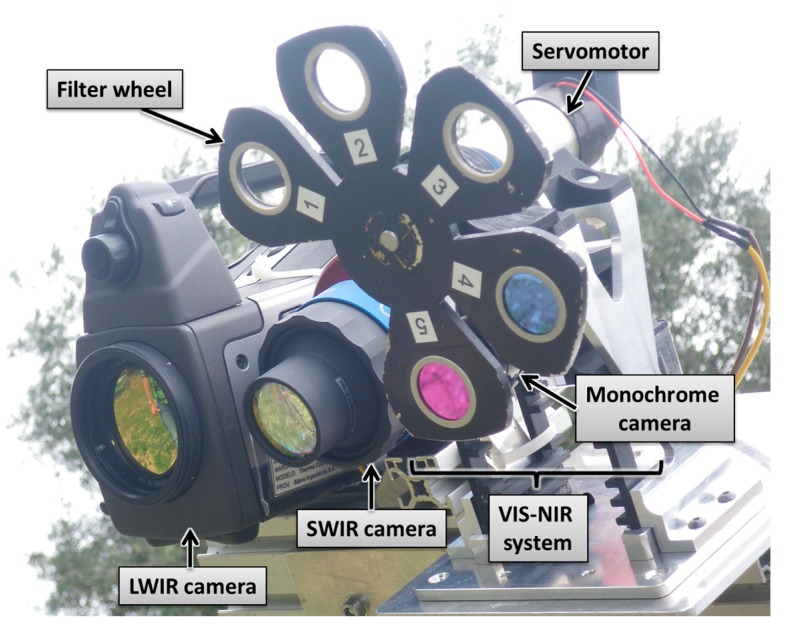
Proposed multisensory system.

The proposed solution is based on the combination of a P25 LWIR camera (FLIR, Wilsonville, OR, USA) a GoldEye P-032 SWIR camera (Allied Vision, Stradtroda, Germany) and a VIS-NIR system that comprises an Allied Vision AVT Prosilica GC2450 high resolution monochrome camera, a custom-made filter wheel, and a servomotor that allows interchanging two band-pass optical filters with centre wavelengths of 624 nm and 950 nm (see Figure 1). The FLIR P25 LWIR camera has a spectral range that goes from 7.5 to 13 μm, a storage temperature range that goes from −40 °C to 70 °C, a thermal sensitivity of 0.08 °C at 30 °C and a resolution of 320 × 240 pixels. The GoldEye P-032 SWIR camera has a spectral response from 900 nm to 1700 nm thanks to its InGaAs sensor of 636 × 508 pixels. The AVT Prosilica GC2450 monochrome camera, which features the high quality ICX-625 CCD image sensor (Sony, Tokyo, Japan), has a frame rate of up to 15 fps at 2448 × 2050 pixels resolution.

Band-pass optical filters with centre wavelengths of 624 nm and 950 nm are selected to enable discrimination between vegetation and ground. It is well documented that all photosynthetic plants are characterised by a low reflectance in red wavelengths (600 nm–700 nm) because chlorophylls (and related pigments) absorb much of the incident energy for photosynthesis. Meanwhile, in the near-infrared wavelengths (700 nm–1300 nm) photosynthesising plants reflect large proportions of the incident sunlight [40,41]. Therefore, processing of these images acquired with the aforementioned filters will allow us to focus only on those pixels of the scene that belong to the ground and that are required for further analysis [42]. Table 2 summarises technical specifications of the selected filters.

**Table 2:** Technical specifications of the selected filters.

FILTER 1		FILTER 2	
Centre wavelength CWL (nm)	624.00	Centre wavelength CWL (nm)	950.00
Bandwidth (nm)	40.00	CWL Tolerance (nm)	±10
Transmitted Wavefront, RMS (λ)	¼	Full Width-Half Max (nm)	50
Bandwidth Tolerance (nm)	±6.24	Full Width-Half Max Tolerance (nm)	±15
Minimum Transmission (%)	90	Minimum Transmission (%)	55
Optical density OD	>6.0	Optical density OD	>3.0

Once these areas of interest have been isolated, they are analysed on SWIR and LWIR images in order to study the surface soil moisture. Water strongly absorbs light in the SWIR wavelengths. This is due to overtones and fundamentals of the three vibration frequencies of H_2_O; symmetric and asymmetric O–H stretching and O–H bending [43,44]. Thus, the reflectance on SWIR images decreases with increasing soil moisture. LWIR thermal images, for its part, show a decrease in temperature in moist soil, in comparison with dry soil [39] due mainly to the evaporation that dissipates heat and the high heat capacity of the water. Nevertheless, there are several factors that can influence soil temperature such as the angle to the sun and the time of day. For that reason, LWIR information is just utilised for determining if the ground exhibits either a heterogeneous or a homogeneous moisture distribution.

**Figure 2:**
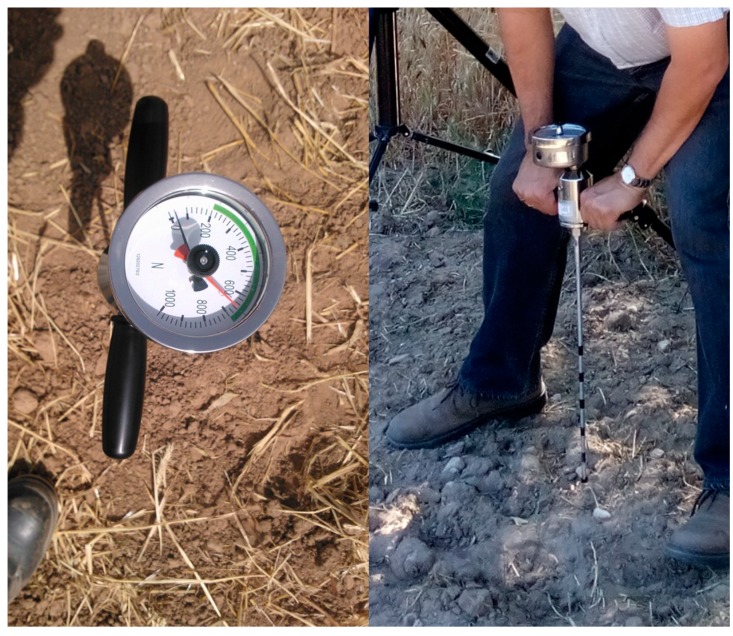
Penetrometer utilised during the experimental tests.

It is also important to mention that during the design phase several measurements were carried out not only with the proposed sensory rig, but also with a penetrometer, so that the soil penetration resistance acquired with the penetrometer could be associated with the normalised reflectance value of the SWIR images. The M06.01 penetrometer (Eijkelkamp, Giesbeek, The Netherlands) utilised during these tests for the acquisition of training data is shown in Figure 2. It is composed of a steel rod fitted with a conical tip, a device to monitor the force, and several marks to locate some predefined positions of the cone. The cone has 60° angle, and the basal area is equal to 1 cm^2^. For each test the cone was pushed into the soil at constant velocity and the penetration resistance was read at certain depths.

Thus, the algorithm proposed for estimating the ground bearing capacity combines:

- A registration procedure. VIS-NIR, SWIR and LWIR images come from cameras that exhibit different field of view and different pixel array. To overcome this problem, the random sample consensus (RANSAC) algorithm [45] is adopted for registering the images acquired with the different cameras that compose the sensory rig, in such way that a direct correspondence between the pixels of the different images is obtained [42]. This enables that following processing steps can be conducted at pixel level.
- A multi-class Support Vector Machine (SVM) classifier that is applied to registered monochrome, 624 nm and 950 nm images and trained to label the pixels of these images into three classes that are: soil, vegetation and others. Two SVMs are executed sequentially, each one for detecting a class against the rest, as described in [42]. The resulting pixel-based classification map is then utilised for generating a mask that will allows us to work only with those pixels that belong to the soil class in the next processing steps and discard the rest of them.
- The masking of the registered SWIR and LWIR images by using the pixel-based classification map obtained in the previous step.
- A K-means clustering that is applied to the soil pixels of the masked thermal image with the aim of partitioning soil pixels into three effective clusters. These three clusters are then utilised for delimiting the areas where the mean temperatures and the mean reflectance values will be calculated from the thermal and the normalised SWIR images, respectively. Next, the absolute differences from the mean values of the clustered areas are calculated and compared with certain predefined thresholds that will help us to determine if the soil sample has a homogeneous bearing capacity, or if on the contrary, the soil is heterogeneous. If soil is homogeneous a unique mean reflectance value is calculated from the soil pixels of the normalised SWIR image. If the soil is heterogeneous, a mean reflectance value is calculated for each area of the normalised SWIR image. Delimitation of these areas is given by the clusters obtained from the masked thermal image after applying the K-means clustering.
- A linear regression that is applied for modelling the relationship between the soil penetration resistance and the mean reflectance value of the areas of interest from the corresponding normalised SWIR image. For accomplish this linear regression, numerous experimental tests were carried out, where the penetration resistance was measured with the penetrometer and the corresponding mean reflectance value was calculated from the normalised SWIR image acquired for the same scenario. Therefore, mean reflectance values obtained in the previous step are used for the final estimation of the ground bearing capacity.

**Figure 3:**
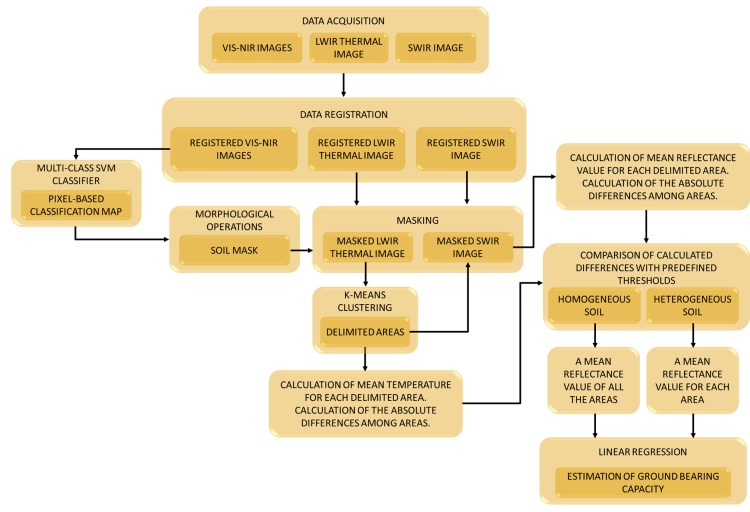
Proposed methodology for estimating the ground bearing capacity.

The methodology proposed for estimating the ground bearing capacity is summarised in Figure 3.

## 3. Experimental Section

Data acquisition was conducted in several campaigns that took place between March and July of 2014, in Madrid, Spain. Sites include CSIC premises and two field stations owned by CSIC at Arganda del Rey (lat. 40°18′48.9″N; long. 03°28′54.8″W), totalling an extension of 64 ha on a typical xerofluvent soil with a sandy-loam texture in the first 0.5 m and increasingly sandy texture below. For a better comprehension, the description of the experimental stage is divided into four main phases that are detailed below.

**Figure 4:**
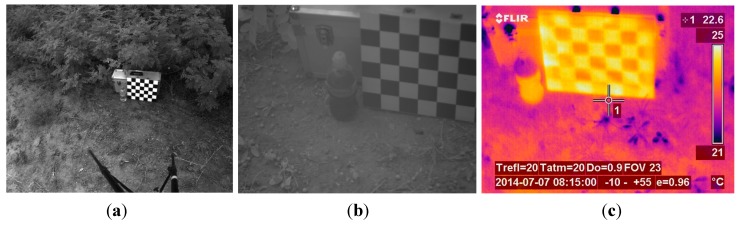
Images utilised as inputs for the registration procedure. (**a**) Monochrome image acquired by the AVT Prosilica GC2450; (**b**) SWIR image; (**c**) LWIR thermal image.

**Figure 5:**
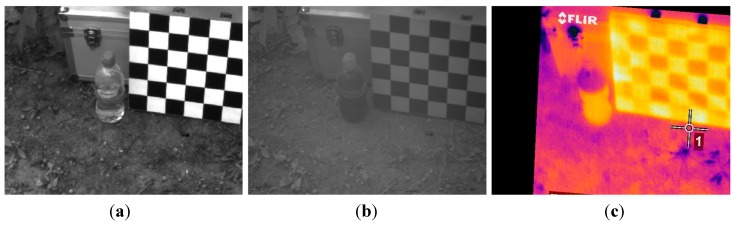
Registered images. (**a**) Registered monochrome image; (**b**) Registered SWIR image; (**c**) Registered LWIR image.

The first phase of the experimental stage was devoted to the acquisition of images for implementing the registration procedure. Although the VIS-NIR, LWIR and SWIR cameras that constitute the proposed multisensory system are utilised for acquiring images from the same scene, resulting images are taken from slightly different viewpoints, with a different field of view and different resolution. Thus, this procedure aims to obtain a direct correspondence between the pixels of the different images before continuing with further processing steps for estimating the ground bearing capacity. The dataset acquired in this first phase included monochrome, LWIR and SWIR images. A chessboard pattern was utilised for facilitating the process of finding the point correspondences. Figure 4 shows an example of the monochrome, LWIR and SWIR images utilised as inputs for the registration procedure, whereas Figure 5 displays the resulting outputs of the aforementioned procedure.

Since in natural scenarios soil is merged with vegetation and other elements, the purpose of the second phase of the experimentation stage was to train and evaluate the multi-class SVM classifier for discriminating the soil from the rest of the scene elements. In this way, only pixels belonging to soil are analysed in LWIR and SWIR imaging during the other processing steps of the proposed algorithm.

**Figure 6:**
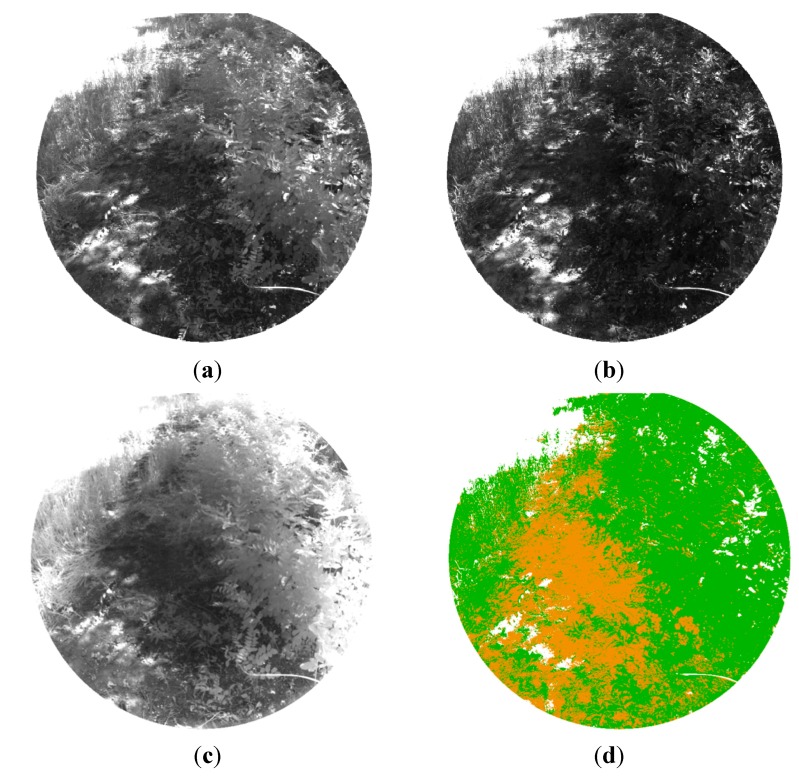
(**a**) Monochrome image; (**b**) 624 nm image; (**c**) 950 nm image; (**d**) Classification map.

For training the SVMs, three acquired dataset were randomly picked. Each dataset acquired in this phase included a monochrome image and two images with band-pass filters that have centre wavelengths of 624 nm and 950 nm. From these monochrome and filtered images, representative regions of interest of different sizes were selected for each desired class. Then, the mean reflectance values of these regions were treated as training samples and were manually labelled in three semantic classes: soil, vegetation and others elements of the scene. With the obtained set of 30 samples per class, the SVMs of the proposed algorithm were trained to classify the pixels of the images. Figure 6 shows an example of the monochrome and the filtered images acquired with the multisensory system for evaluating the proposed multi-class SVM classifier, as well as the resulting classification map. In the classification map, brown, green and white colours are utilised to visualize pixels classified as soil, vegetation and other elements, respectively.

**Figure 7:**
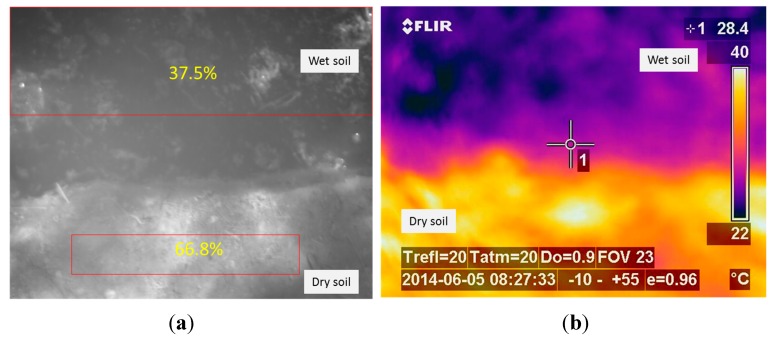
(**a**) Normalised SWIR image; (**b**) LWIR thermal image.

**Figure 8:**
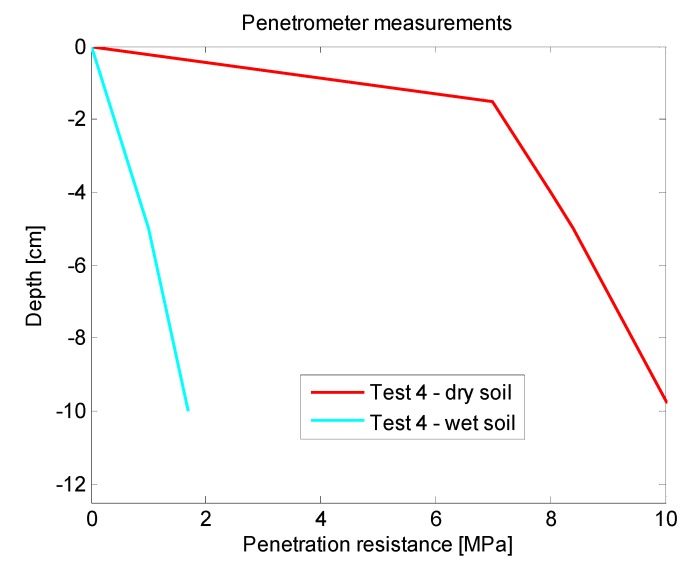
Soil penetration resistances acquired with the penetrometer.

The third phase of the experimental stage was carried out with the aim of finding out a relationship between the data acquired with the SWIR camera and the measurements obtained with the penetrometer. Datasets included LWIR and SWIR images acquired in scenarios with dry and wet soil. Moreover, in each of the conducted experiments, several measures were also carried out with a penetrometer, so that the soil penetration resistance information can be associated with the corresponding mean reflectance value of the SWIR image acquired with the sensory rig, and in this way, be subsequently used for training the algorithms responsible of estimating the ground bearing capacity. An example of the data obtained in this phase is presented on Figure 7 and Figure 8. Figure 7a shows the normalised image acquired with the SWIR camera, while Figure 7b illustrates the corresponding LWIR image. Note that upper parts of these images correspond to wet soil and bottom parts to dry soil. Red boxes displayed on Figure 7a indicate the dry and wet areas of the soil that were selected not only for the calculation of the mean reflectance percentages but also for the measurement of the penetration resistances by using the penetrometer. Resulting mean reflectance percentages for the selected areas are also displayed in yellow. Figure 8 gathers the measurements acquired with the penetrometer for this test. Black and blue lines represent the penetration resistances measured at different depths when the soil is dry and wet, respectively.

**Figure 9:**
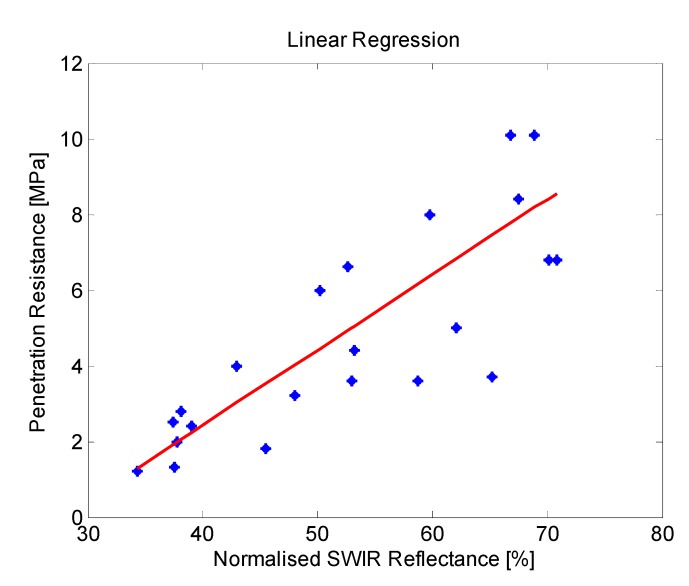
Relationship between soil penetration resistance and normalised SWIR reflectance.

From the results obtained in all the tests carried out on this third phase it is possible to confirm that the mean reflectance values of the areas of interest belonging to the soil on normalised SWIR images decrease with increasing soil moisture, as it was expected, since water strongly absorbs light in the SWIR wavelengths. Thermal images also confirm a decrease in the temperature through the day when the ground is wet, in comparison with the dry soil. However, as we stated in the previous section, the direct utilisation of thermal information is discarded for the estimation of the ground bearing capacity, since there are several factors that influence soil temperature, including angle to the sun, weather conditions and the time of day. Even so, thermal information is very useful for other intermediate processing steps that help us to identify areas that are considerably wetter than the rest, providing automatic delineation of areas for the calculation of the mean reflectance values on normalised SWIR images. On the other hand, penetrometer measurements reinforce the idea that the penetration resistance, and therefore, the bearing capacity, decrease when the soil is moist. Taking into account these results, a linear regression approach is applied for modelling the relationship between the soil penetration resistance (at a depth of 0.1 m) and the corresponding mean reflectance value of this region of interest on a normalised SWIR image. Figure 9 illustrates the linear regression carried out with the data obtained from the experimental tests.

Final set of experiments were intended to evaluate the whole system proposed for the estimation of the ground bearing capacity. Acquired datasets included LWIR thermal, SWIR and monochrome images, as well as two filtered images acquired with band-pass filters that have centre wavelengths of 624 nm and 950 nm. Results of these experiments are described in next Section.

## 4. Results and Discussion

**Figure 10:**
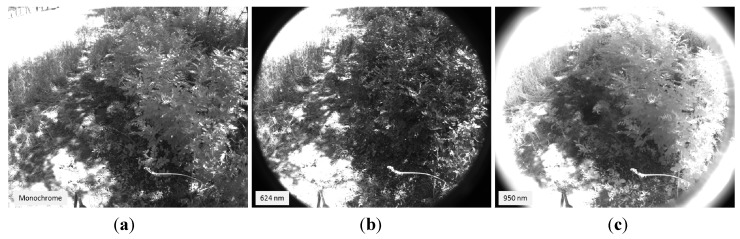
(**a**) Monochrome image; (**b**) 624 nm image; (**c**) 950 nm image.

**Figure 11:**
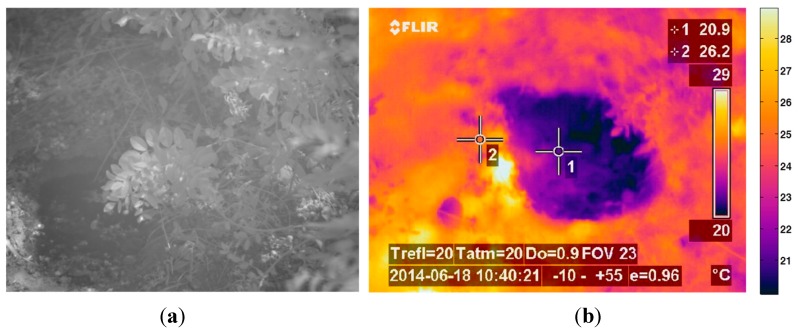
(**a**) Normalised SWIR image; (**b**) LWIR thermal image.

**Figure 12:**
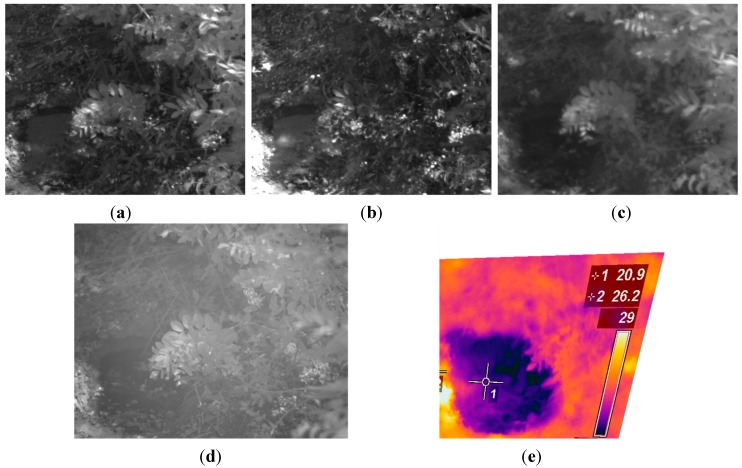
Registered images. (**a**) Registered monochrome image; (**b**) Registered 624 nm image; (**c**) Registered 950 nm image; (**d**) Registered SWIR image; (**e**) Registered LWIR image.

**Figure 13:**
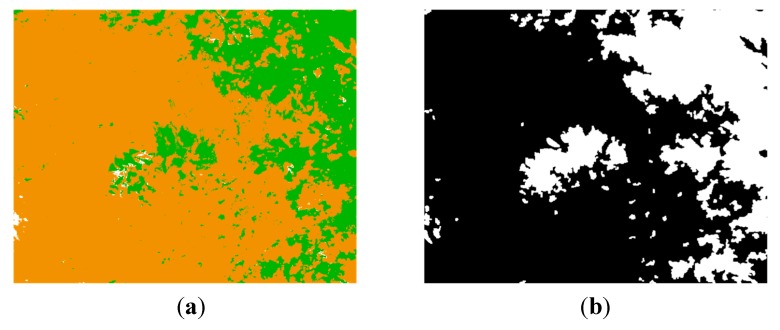
(**a**) Classification map; (**b**) Mask generated from the classification map.

**Figure 14:**
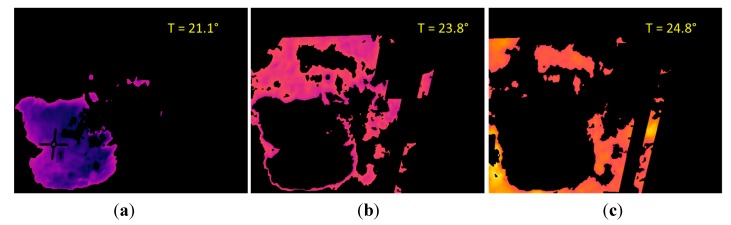
Effective clusters resulting from the K-means procedure. (**a**) First cluster, named C1; (**b**) Second cluster; (**c**) Third cluster.

**Figure 15:**
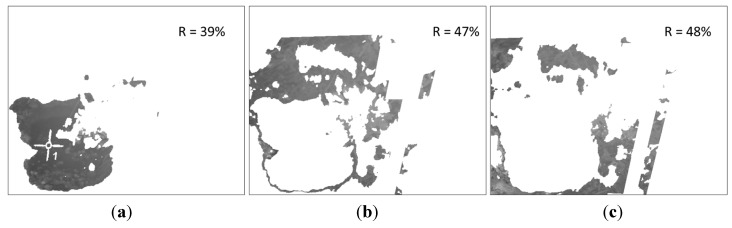
Delimited areas and mean reflectance percentages on the normalised SWIR image. (**a**) First cluster; (**b**) Second cluster; (**c**) Third cluster.

In order to validate the capabilities of the proposed system, a total of 10 scenes with variable soil water content conditions were acquired, processed and evaluated. Ground truth data was carefully collected with a penetrometer for each scene in order to carry out a quantitative assessment of the proposed solution. Figure 10, Figure 11, Figure 12, Figure 13, Figure 14 and Figure 15 illustrate most of the intermediate results obtained from the different steps that make up the algorithm proposed for the estimation of the ground bearing capacity. Figure 10 and Figure 11 display the dataset of a scene acquired with the proposed multisensory system. This dataset includes a monochrome image, two filtered images acquired with band-pass filters whose centre wavelength are 624 and 950 nm, a normalised SWIR image and a LWIR thermal image.

Figure 12 shows the resulting images after applying the registration procedure. This pre-processing step allows us to have a direct correspondence between the images acquired with the different cameras, enabling the ground bearing estimation algorithm to operate at pixel level.

The pixel-based classification map resulting from applying the multi-class SVM classifier to the images presented in Figure 12a–c is shown in Figure 13a. Note that in spite of the challenging scene, the multi-class SVM classifier exhibits a very successful performance. This map is then utilised for generating a mask, in such a way that only pixels classified as soil are considered on thermal and normalised SWIR images for the further processing steps. Thus, Figure 13b displays the mask generated from the classification map and after the application of some morphological procedures to fill holes and remove small objects.

Next, the K-means clustering is applied to the pixels of the LWIR thermal image that remain after conducting the masking, with the aim of partitioning these pixels into four exclusive clusters. Every pixel in the image is labelled in accordance with the cluster index assigned by the K-means procedure. Cluster corresponding to white pixels is disregarded, since it does not belong to the soil class. Figure 14 shows the three effective clusters resulting from the K-means procedure. These three effective clusters are then utilised for delimiting the areas that are employed for calculating the mean temperatures and the mean reflectance values on the thermal and the normalised SWIR images, respectively. Figure 15 displays the corresponding delimited areas on the normalised SWIR image.

The comparison of the absolute differences obtained from the different clusters with certain predefined values, will help us to determine if the soil sample has a homogeneous bearing capacity, or if on the contrary, the soil is heterogeneous, exhibiting areas with significantly different bearing capacities (for instance, due to an area with a greater water content). This last case is clearly observed in Figure 14 and Figure 15, where the first cluster, named C1, presents a mean temperature (Figure 14a) and a mean reflectance value (Figure 15a) notably different from the other clusters. It is important to mention that on the proposed algorithm two conditions have to be fulfilled simultaneously so that two clusters can be considered as independent: the absolute difference of the mean temperatures must be greater than one degree and the absolute difference of the mean reflectance percentages must be greater than five points. These thresholds have been empirically defined. Therefore, in the stated example, the ground bearing capacity is estimated for two areas: the first one provided by the C1 cluster, and the second one provided by the two other clusters.

Then, taking into account these estimations, the model obtained from the linear regression presented in Figure 9 is applied, resulting in a ground bearing capacity of 2.22 MPa for the region provided by the cluster C1 and 3.92 MPa for the area provided by the other two clusters. Ground truth data obtained with the penetrometer indicated a ground bearing capacity of 2.4 MPa for the first region and 4 MPa for the second one, resulting in a mean relative error of 4.75% in the estimation of the ground bearing capacity for this test.

**Figure 16:**
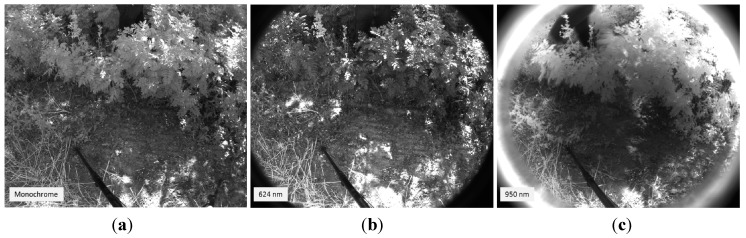
(**a**) Monochrome image; (**b**) 624 nm image; (**c**) 950 nm image.

**Figure 17:**
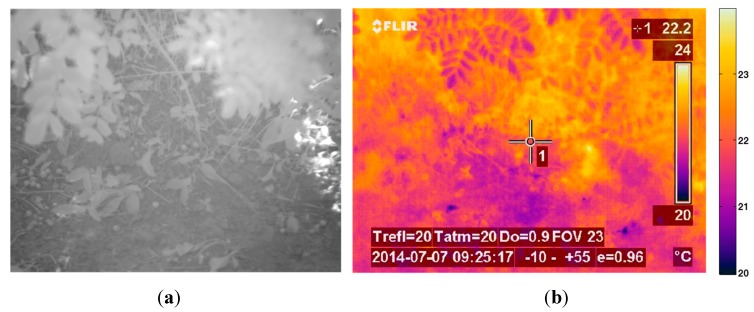
(**a**) Normalised SWIR image; (**b**) LWIR thermal image.

**Figure 18:**
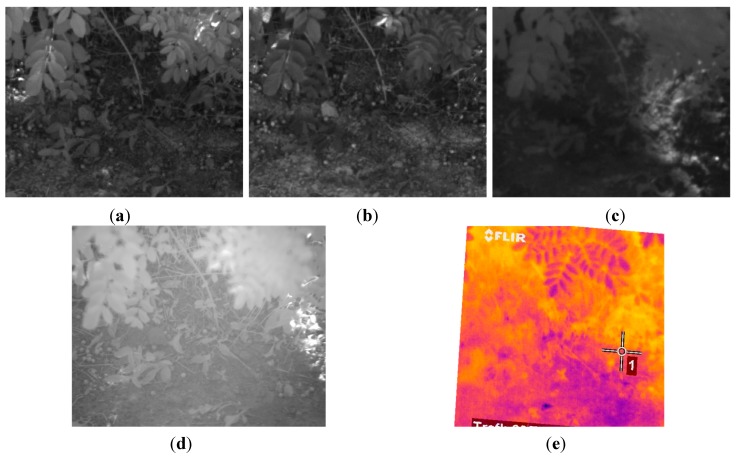
Registered images. (**a**) Registered monochrome image; (**b**) Registered 624 nm image; (**c**) Registered 950 nm image; (**d**) Registered SWIR image; (**e**) Registered LWIR image.

**Figure 19:**
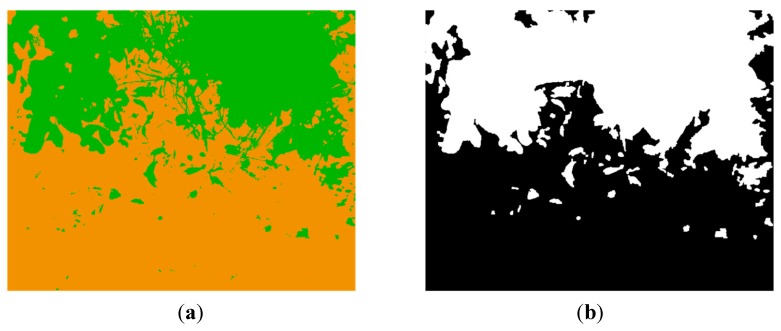
(**a**) Classification map; (**b**) Mask generated from the classification map.

**Figure 20:**
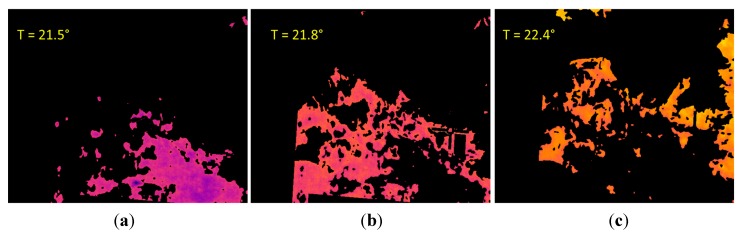
Effective clusters resulting from the K-means procedure. (**a**) First cluster; (**b**) Second cluster; (**c**) Third cluster.

**Figure 21:**
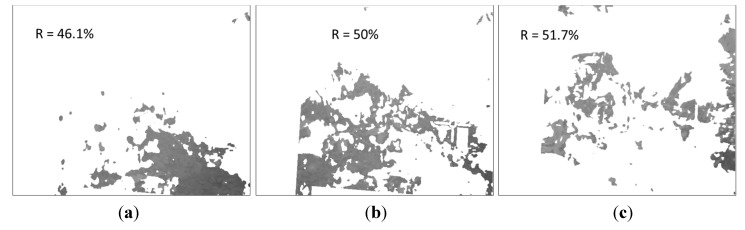
Delimited areas and mean reflectance percentages on the normalised SWIR image. (**a**) First cluster; (**b**) Second cluster; (**c**) Third cluster.

Figure 16, Figure 17, Figure 18, Figure 19, Figure 20 and Figure 21 depict the same intermediate steps and results described above for an additional scene characterised for exhibiting a different behaviour of the ground bearing capacity. Figure 16 and Figure 17 display the dataset of the scene acquired with the proposed multisensory system. This dataset includes a monochrome image, two filtered images acquired with band-pass filters whose centre wavelength are 624 and 950 nm, a normalised SWIR image and a thermal image.

Figure 18 shows the images obtained after applying the registration procedure. Figure 19 presents the map resulting from the multi-class SVM classifier and the mask generated from this classification map.

Next, Figure 20 shows the three effective clusters resulting from the K-means procedure, while Figure 21 displays the corresponding delimited areas on the normalised SWIR image. Since in this case the absolute differences of the mean temperatures of the clusters are less than or equal to one degree, they are considered as a unique cluster with a homogeneous ground bearing capacity. Then the proposed algorithm recalculates the mean reflectance percentage for the whole area and provides an estimated ground bearing capacity of 4.3 MPa. Ground truth data obtained with the penetrometer indicated a mean ground bearing capacity of 4.0 MPa for this region of interest, resulting in a mean relative error of 7.5% in the estimation of the ground bearing capacity for this test.

LWIR thermal images acquired during the experimental tests demonstrated that under nominal weather conditions, wet soil is cooler than dry soil through the day. At both high and low temperatures, there was still a notably thermal difference between the dry and wet soil. For that reason, K-means clustering applied to the masked thermal images resulted to be a suitable option to delimit areas with different temperatures, and consequently, areas that are candidates to present different water content. However, temperature values are disregarded for the direct estimation of the ground bearing capacity due to its high variability depending on several external factors. Results from the clustering procedure are also applied to the normalized SWIR images. This is a great advantage, since although mean reflectance values on normalized SWIR images diminish with increasing soil water content, differences in a heterogeneous terrain can be difficult to detect. On the other hand, the multi-class SVM classifier applied to the VIS-NIR images also demonstrated to be very useful for discriminating soil from other elements of the scene. Discarding those pixels that belong to other elements of the scene different from soil contributes to reduce estimation errors during the calculation of the mean reflectance values from the SWIR images. Despite of the successful performance of the classifier, a closer observation of the results shown in Figure 13a and Figure 19a brings into relief that some common misclassification errors can be produced due to shadows and white bright pixels from overexposed areas. However, the effects of these misclassifications are minimised thanks to the subsequent steps of the proposed algorithm. For instance, white bright pixels are usually classified as other elements of the scene. Therefore, these pixels will be discarded for further analysis, contributing in this way to reduce ground bearing capacity estimation errors. The same happens if shadowed pixels are classified as vegetation or as other elements of the scene. On the contrary, if shadowed pixels are classified as belonging to the class soil, a more complex analysis is required to determine its influence in the estimation of the ground bearing capacity. Nevertheless, in the worst case, the proposed approach is still quite conservative, since shadows would produce areas with low reflectance values in SWIR imagery that would tend to decrease the magnitude of the ground bearing capacity. This result is still beneficial from the point of view of the soil protection. Thus, combination of LWIR, SWIR and VIS-NIR imagery through the proposed algorithm minimises the disadvantages and maximises the strengths of each independent system, resulting in an effective and non-invasive solution for estimating the ground bearing capacity.

Gathering the results obtained from the 10 evaluated scenes, it is possible to highlight that the proposed approach showed a capability of 79.3% for predicting correctly the ground bearing capacity from the mean reflectance value of the normalized SWIR images, with a mean prediction error of 20.7%. Despite the high level of correctness attained by the proposed solution when it comes to estimating the ground bearing capacity, it should be taken into account that the prediction model obtained from the linear regression mainly relies on experimental data. Therefore, in order to guarantee a robust estimation of the ground bearing capacity, data acquisition should be extended to different types of terrains. Even, it could be desired to count with one predictive model for each type of terrain.

The presented approach can be also very useful for helping decision making systems in selecting those areas that tend to minimize ground damage during mechanical operation with wheeled mobile robots or vehicles.

## 5. Conclusions and Future Research Directions

This paper proposes a multisensory system based on VIS-NIR, SWIR and LWIR imagery and a sequential algorithm that combines a registration procedure, a multi-class SVM classifier, a K-means clustering and a linear regression for estimating the ground bearing capacity. The presented solution can be utilized in natural scenarios, in real-time and in a non-destructive manner. Therefore, the sensory rig and the cited algorithm can be installed on-board a mobile robot or vehicle for estimating ground conditions before traversing the field, avoiding disturbances of the site and significantly reducing ground damages.

An extensive experimental campaign was carried out not only for acquiring the data required for the design phase but also for assessing the capabilities of the proposed approach. Experimental results have shown that both the multisensory system and the sequential algorithm exhibit a satisfactory performance. Comparison of ground bearing capacity estimations resulting from the experimental tests with the ground truth data obtained with the penetrometer shows the feasibility and the potential of the proposed method. Future work should be directed to enhancing the prediction model obtained from the linear regression. For that, a more widespread experimental study should be conducted in order to include different types of soils that contribute to guarantee a robust estimation of the ground bearing capacity.
